# Profiling plasma‐extracellular vesicle proteins and microRNAs in diabetes onset in middle‐aged male participants in the ELSA‐Brasil study

**DOI:** 10.14814/phy2.14731

**Published:** 2021-02-15

**Authors:** Laureane N. Masi, Paulo A. Lotufo, Frederico M. Ferreira, Alice C. Rodrigues, Tamires D. A. Serdan, Talita Souza‐Siqueira, Aécio A. Braga, Magda E. G. Saldarriaga, Tatiana C. Alba‐Loureiro, Fernanda T. Borges, Diego P. Cury, Mario H. Hirata, Renata Gorjão, Tania C. Pithon‐Curi, Simão A. Lottenberg, Ligia M. G. Fedeli, Helder T. I. Nakaya, Isabela J. M. Bensenor, Rui Curi, Sandro M. Hirabara

**Affiliations:** ^1^ Interdisciplinary Post‐graduate Program in Health Sciences Cruzeiro do Sul University Sao Paulo Brazil; ^2^ Center for Clinical and Epidemiologic Research University of Sao Paulo Sao Paulo Brazil; ^3^ Department of Pathology University of Sao Paulo School of Medicine Sao Paulo Brazil; ^4^ Department of Pharmacology Instit ute of Biomedical Sciences University of Sao Paulo Sao Paulo Brazil; ^5^ Faculty of Pharmaceutical Sciences University of São Paulo Sao Paulo Brazil; ^6^ Department of Anatomy Institute of Biomedical Sciences University of Sao Paulo Sao Paulo Brazil; ^7^ Faculty of Medicine University of Sao Paulo Hospital das Clínicas Sao Paulo Brazil; ^8^ Butantan Institute São Paulo Brazil

**Keywords:** epigenetics, glucose intolerance, hyperglycemia, proteomic

## Abstract

We measured plasma‐derived extracellular vesicle (EV) proteins and their microRNA (miRNA) cargos in normoglycemic (NG), glucose intolerant (GI), and newly diagnosed diabetes mellitus (DM) in middle‐aged male participants of the Brazilian Longitudinal Study of Adult Health (ELSA‐Brazil). Mass spectrometry revealed decreased IGHG‐1 and increased ITIH2 protein levels in the GI group compared with that in the NG group and higher serotransferrin in EVs in the DM group than in those in the NG and GI groups. The GI group also showed increased serum ferritin levels, as evaluated by biochemical analysis, compared with those in both groups. Seventeen miRNAs were differentially expressed (DEMiRs) in the plasma EVs of the three groups. DM patients showed upregulation of miR‐141‐3p and downregulation of miR‐324‐5p and ‐376c‐3p compared with the NG and GI groups. The DM and GI groups showed increased miR‐26b‐5p expression compared with that in the NG group. The DM group showed decreased miR‐374b‐5p levels compared with those in the GI group and higher concentrations than those in the NG group. Thus, three EV proteins and five DEMiR cargos have potential prognostic importance for diabetic complications mainly associated with the immune function and iron status of GI and DM patients.

## 1 INTRODUCTION

Diabetes is a major risk factor for all‐cause deaths and disability worldwide (Williams & Loeffler, 2019). Diabetes complications occur due to long‐term hyperglycemia exposure, even after a tight glycemic control regimen (Pirola et al., 2010), termed the metabolic memory or legacy effect (Chalmers & Cooper, 2008). Novel biomarkers for new‐onset diabetes and diabetic complications might reduce the health‐adverse outcomes.

Extracellular vesicles (EVs) and their cargos act as conduits for cell‐to‐cell communication (Lotvall & Valadi, 2007) and are potential biomarkers for the early diagnosis of various diseases, such as diabetes (Sáez et al., 2019), neurodegenerative diseases (Li et al., 2018), cancer (Louis et al., 2019), and hypertension (Hogan et al., 2019). EVs are a heterogeneous population of phospholipid bilayered membrane vesicles secreted into the extracellular space by several cell types in both healthy and diseased states (Yáñez‐Mó et al., 2015). Typically ranging from 30 to 1,500 nm in diameter, depending on their origin, EVs carry several macromolecules, such as proteins, lipids, microRNAs, mRNAs and mitochondrial DNA (Greening et al., 2017; Valadi et al., 2007). In disease states and even under physiological conditions, changes in the peripheral blood occur not only in the quantity and subpopulation of extracellular vesicles but also in those of the protein and microRNA cargos. The specific plasma miRNA expression profile can be used as the fingerprint of a physiological or disease condition (Chen et al., 2008).

MicroRNAs (miRNAs) are endogenously expressed, evolutionary conserved, small single‐stranded, noncoding RNA molecules of 21–23 nucleotides. The molecules regulate gene expression by destabilizing or inhibiting target mRNA translation by base‐pairing to the 3′ untranslated regions (Bartel, 2004). Circulating miRNAs are postulated as novel biomarkers to be used as reflective or predictive indicators of metabolic health and disorders (Karolina et al., 2011). MiRNAs can be released into body fluids by different cell types and can be transported free or inside extracellular vesicles to other cells (Lorente‐Cebrián et al., 2019). Blood miRNAs are potential biomarkers to predict DM onset and associated complications (Chien et al., 2015; Guay & Regazzi, 2013). Zampetaki et al. (2010) revealed distinct free serum miRNA profiles in patients with DM compared with non‐DM patients in a Bruneck cohort study using miRNA microarrays. Karolina et al. (2012) reported similar findings in a Singapore cohort. The candidate miRNAs are mainly involved in the regulation of insulin secretion, insulin resistance, glucose homeostasis, and lipid metabolism associated with DM pathology (Chen et al., 2008; Dehwah et al., 2012; Jeon et al., 2014; Karolina et al., 2011). However, the miRNAs involved in the etiology and pathogenesis of DM remain to be completely elucidated. Inconsistencies exist in the results concerning DM‐associated miRNAs possibly due to ethnic variations, differences in inclusion/exclusion criteria, methods of miRNA analysis, and their association with proteins, lipids, and EVs (Chen et al., 2008; Dehwah et al., 2012; Jeon et al., 2014; Karolina et al., 2011). As mentioned above, the analysis of plasma EVs and their cargos is promising to discover novel biomarkers for diabetes onset or the management of diabetic complications. In the present study, we investigated the differences in plasma EV proteins and miRNA cargo profiling in normoglycemic, glucose‐intolerant, and newly diagnosed DM middle‐aged male participants, without previous clinical manifestations, from the Brazilian Longitudinal Study of Adult Health (ELSA‐Brasil) (Aquino et al., 2012; Lotufo, 2013; Schmidt et al., 2014). This type of DM is generally manifested in both sexes after the age of 40 years and is regarded as a typical age‐related disease (Vaiserman & Lushchak, 2019). However, specific miRs in circulating EVs differ in terms of the age of the woman and estrogen‐based hormone replacement therapy, suggesting that systemic estrogen levels affect the miRNA profile (Kangas et al., 2017). Based on this, our investigation in the present study mainly involved plasma samples from males.

## 2 MATERIAL AND METHODS

### 2.1 Samples

The ELSA‐Brasil is a multicenter study designed to investigate the incidence of diabetes and cardiovascular diseases and their biological, behavioral, environmental, occupational, psychological, and social risk factors (Aquino et al., 2012; Lotufo, 2013; Schmidt et al., 2015). Briefly, active or retired civil servants (aged 35–74 years) from six Brazilian cities (Belo Horizonte, Porto Alegre, Rio de Janeiro, Salvador, São Paulo, and Vitoria) were eligible for the cohort study. The first examinations of 15,105 individuals were carried out from August 2008 through December 2010. The ethics committee of all institutions approved the research protocol, and all participants signed written consent. The present analysis is a cross‐sectional study using a subsample of participants from the ELSA‐Brasil site Sao Paulo. A random sample of 62 male individuals, middle‐aged, overweight, without previous diabetes and cardiovascular disease, was selected from a total of 5,061 participants from the ELSA‐Brasil in Sao Paulo. We distributed the participants into three groups based on the presence of at least one of the following criteria: fasting plasma glucose, plasma glucose after the oral glucose tolerance test and glycated hemoglobin measurements (individual data are available in the Appendix S1) following the American Diabetes Association guidelines (Association, 2018). The groups were as follows: normoglycemia (NG), glucose intolerant (GI), and newly diagnosed with diabetes at the baseline examination (DM). After nocturnal fasting, the blood samples were collected from the participants in EDTA‐treated tubes (plasma separation) or tubes containing clot activator gel (serum separation). The samples were centrifuged for 15 min at 1,500 ***g*** and room temperature, and plasma and serum were stored in sterile tubes at −80°C until analysis. Hemolysis in serum and plasma samples was assessed by simple visual inspection for pink discoloration indicative of free hemoglobin against a white background with the possibility of detection down to 0.25% (Shah et al., 2016). The exclusion factors were as follows: the use of medications for diabetes or prediabetes, a history of severe muscle injury, endocrine‐related disorders or the use of hormonal/nutritional supplements.

### 2.2 Laboratory measurements

All the measurements were performed at the University Hospital of the University of Sao Paulo, as previously described (Fedeli et al., 2013). Serum transferrin was analyzed by direct determination using the Ferrozine method performed on the Roche Cobas c501 automated equipment. Under acidic conditions, iron is released from transferrin and the color intensity is directly proportional to the concentration of the unbound iron excess and inversely proportional to the unsaturated iron binding capacity. Absorbance was determined at 700/546 nm.

### 2.3 Plasma‐derived extracellular vesicle isolation

The extracellular vesicles were separated from plasma samples (approximately 600 µl) using the miRCURY Exosome Isolation Kit‐Serum and Plasma (Exiqon) following the manufacturer's instructions. Briefly, after thawing the samples on ice, residual cells, debris, platelets, and apoptotic bodies were pelleted by centrifugation (5 min at 10,000 ***g*** at room temperature) and the supernatant was transferred to a sterile tube. The sample was mixed with the precipitation buffer (200 µl) provided by the kit and then was incubated for 60 min at 4°C. The sample was centrifuged (5 min at 500 ***g*** at room temperature) to pellet the extracellular vesicles. The supernatant was discarded, and the isolated extracellular vesicles were resuspended (300 µl) for EV characterization and miRNA extraction. Random plasma samples (*n* = 4) from health volunteers were used to standardize the EV isolation as described below (items 2.3.1, 2.3.2, and 2.3.3).

#### 2.3.1 Characterization of the extracellular vesicles by transmission electron microscopy

The isolated extracellular vesicles resuspended in 300 µl were fixed in 2% formaldehyde (Dinâmica #2047) for 2 h at 4°C, followed by the addition of 5 µl of the suspension onto Formvar‐carbon‐coated EM grids (EMS #FCFT200‐Cu). The grids were covered, and the membranes were absorbed for 20 min in a dry environment. We added PBS (100 µl) on a parafilm sheet. The grids with the membrane side down were placed over a drop for 1 min to wash. The grids were transferred to 50 µl of 1% glutaraldehyde (Sigma‐Aldrich #G5882) for 5 min and then to 100 µl of distilled water for 2 min. This step was repeated seven times. For contrast, the grids were placed over 50 µl of 4% uranyl acetate (EMS, #22400) for 7 min and examined the next day under a transmission electron microscope (Tecnai FEI G20 at 200 kV) at the Microscopy Center of the Department of Development and Cell Biology of the Institute of Biomedical Sciences of the University of Sao Paulo.

#### 2.3.2 Distribution of isolated EVs by size

Analysis of the absolute size distribution was performed using NanoSight LM10 with NTA2.3 (NanoSight Ltd.). The particles were automatically tracked and sized based on Brownian motion and the diffusion coefficient. After isolation, the plasma EVs were resuspended in 0.5 ml of nanopure water. The NTA measurement conditions were as follows: temperature of 21.0 ± 0.5°C; viscosity of 0.99 ± 0.01 cP, frames per second of 25, measurement time of 30 s, camera level of 11 (NTA 3.0 levels) and Threshold of 3. The detection threshold was similar in all samples. Three recordings were performed for each sample.

#### 2.3.3 Evaluation of extracellular vesicle markers by western blotting

The protein concentration of isolated extracellular vesicles in 300 µl was determined using the Pierce BCA Protein Assay Kit (Thermo Scientific). The samples were lysed with RIPA reagent (Millipore) containing a protease inhibitor cocktail (Roche, Mannheim, Germany) and 10% 0.1 M PMSF (phenylmethyl sulphonyl Fluoride, Sigma‐Aldrich). The proteins were denatured in SDS‐PAGE sample buffer by heating at 95°C for 15 min. Next, 40‐μg protein samples were subjected to 12% SDS‐PAGE, followed by transfer to membranes. The blots were incubated with the primary antibodies anti‐CD9, anti‐CD81, and anti‐HSP70, followed by incubation with rabbit anti‐Ig secondary antibodies (SBI System Biosciences). The specific bands were detected using the ECL chemiluminescent substrate (GE Healthcare Life Science) and then were visualized using the Amersham Imager 600 imaging system (GE Healthcare).

### 2.4 Mass spectrometry, database search, and data processing

The EVs were precipitated from the plasma samples of the three groups (*n* = 7 per group) as mentioned above. The protein concentration was determined as cited above following the protocol described by Kawahara et al. (Kawahara et al., 2017). Briefly, 10‐µg samples were treated with 1.6 M urea, following reduction (5 mM dithiothreitol, 25 min at 56°C), alkylation (14 mM iodoacetamide, 30 min at room temperature in the dark), and digestion with trypsin (1:50, w/w; Promega) at 37°C for 14 h. The reaction was stopped with 1% trifluoroacetic acid (TFA) and desalted with stage tips. The samples were dried in a vacuum concentrator and reconstituted in 0.1% formic acid. For protein analysis, 4.5 µL of the digested proteins was separated using a C18 (100 mm^6^) RP‐nano UPLC (nanoAcquity; Waters) coupled to a Q‐Tof Premier mass spectrometer (Waters) with a nanoelectrospray source at a flow rate of 0.6 ml/min. The gradient was 2%–90% acetonitrile in 0.1% formic acid over 45 min. The nanoelectrospray voltage was 3.5 kV, the cone voltage was 30 V, and the temperature was 100°C. The machine was operated in the ‘top three’ mode, in which one MS spectrum is acquired followed by MS/MS of the top three most‐intense peaks detected. After MS/MS fragmentation, the ion was on the exclusion list for 60 s. We used a real‐time exclusion system to analyze the endogenous cleaved peptides. The spectra were acquired using MassLynx v.4.1 software (https://waters‐masslynx‐scn781.software.informer.com/4.1/), and the raw data files were converted to a peak list format (.mgf) without summing the scans using the Mascot Distiller v.2.3.2.0, 2009 software (https://www.matrixscience.com/distiller.html *)* (Matrix Science Ltd.). The protein list was searched against the UniProt human database (92,180 sequences, 36,693,332 residues; released in March of 2016) using Mascot engine v.2.3.01 (https://www.matrixscience.com/) (Matrix Science Ltd.), with carbamidomethylation as the fixed modification, oxidation of methionine as the variable modification, one trypsin missed cleavage and a tolerance of 0.1 Da for both the precursor and fragment ions. The results were normalized and compared by one‐way ANOVA (*p* < .05) using the Scaffold 3.4 program. The Scaffold v.4.11.01 software (http://www.proteomesoftware.com/products/scaffold/) was defined with the Scaffold Confidence Filters (1) Protein threshold (99%) and (2) Peptide threshold (95%). Only proteins identified by at least one sample that found at least one filter were reported.

### 2.5 Protein data handling and presentation

Functional‐enrichment analysis for Gene Ontology terms was performed using EVpedia (v2.0; http://student4.postech.ac.kr/evpedia2_xe/xe/) (Choi et al., 2013, 2015; Kim et al., 2013). Gene ontology (GO) analysis of annotated proteins was performed for cellular components, molecular function, and biologic processes as previously described (Kim et al., 2013). Enriched terms were ranked by *p*‐value (hypergeometric test) using EVpedia. Comparison of identified proteins with the previously published data was performed using the dataset from the EVpedia online database.

### 2.6 Microarray allows the measurement of the expression profile of EV microRNA

RNA isolation and microarray gene expression profiling were performed by “Exiqon Services” in Vedbaek, Denmark. Forty‐five plasma samples (*n* = 15 per group) were sent to the Exiqon company in Denmark. The ten samples from each group with better quality after the extraction were evaluated in a microarray. The quality of the RNA extraction was determined including three RNAs spike‐ins (UniSp2, UniSp4, UniSp5) during purification step and testing them for the expression after reverse transcribed into cDNA. Total RNA was extracted from the plasma‐derived extracellular vesicles using the miRCURY RNA Isolation kit‐Biofluids (Exiqon). An RNA spike‐in template mixture (UniSp2 and UniSp4) was added to the samples. Reverse transcription of RNA was performed using the miRCURY LNA Universal RT microRNA PCR, Polyadenylation, and cDNA synthesis kit (Exiqon). Each RT was performed including an artificial RNA spike‐in (UniSp6). The cDNA was analyzed by PCR according to the protocol for the miRCURY LNA Universal RT microRNA PCR kit. Each sample was evaluated using the 384‐well real‐time polymerase chain reaction (RT–PCR) method of the microRNA Ready‐to‐Use PCR panel, Custom Pick and Mix Panel (Exiqon), with 185 microRNAs, using The ExiLENT SYBR Green master mix (ExiQon). Negative controls were similarly processed. Amplification was performed using the LightCycler 480 Real‐Time PCR System (Roche) in 384‐well plates. The amplification curves were analyzed using Roche LC v.1.5 software (https://lifescience.roche.com/en_br/products/lightcycler14301‐480‐software‐version‐15.html) to determine the cycle quantification (Cq). Only the samples detected with 5 Cq values smaller than the negative control and with Cq < 37 were included in the analyses. The normalization was performed using the mean values of Cqs of the miRNAs, determined in all samples, as recommended as the best normalizer for qPCR array studies (Mestdagh et al., 2009). In the present study, 85 miRNAs were included in the mean to normalize the Cq values using the following formula: Normalized Cq = global mean Cq−assay Cq (sample).

### 2.7 MicroRNA data handling and presentation

To predict genes targeted by the differentially expressed miRNAs (DEMiRs), the miRWalk 2.0 database (www.umm.uni‐heidelberg.de/apps/zmf/mirwalk/) was used (Dweep & Gretz, 2015). Using the Kyoto Encyclopedia of Genes and Genomes (KEGG), pathway enrichment analysis was performed for the genes targeted by the DEMiRs (Kanehisa & Goto, 2000). The functional regulations between the DEMiRs were assembled and visualized using Cytoscape software (http://www.cytoscape.org/) (Shannon et al., 2003). This network is a graph containing a set of vertices (nodes) corresponding to miRNAs. A directed edge (connection) from an miRNA to another exists if the same targets integrate their relationship. Next, we calculated the degree of the network defined as the number of neighbors (edges) and thickness (targets) of a node. The nodes with a higher degree of centrality are considered biologically relevant miRNAs within biological networks. All the edges are supported by at least ten targets, and the thickness directly represents the number of targets.

### 2.8 Statistical analyses

The clinical and anthropometric data of the participants were presented as means ± SEM and were analyzed by one‐way ANOVA and Tukey's post hoc test in the GraphPad 7.0 program.

The miRNAs detected in the microarray were statistically analyzed to identify the DEMiRs, fitting the expression data to the linear model used in the R/Bioconductor package LIMMA (Ritchie et al., 2015). We used independent 2‐class *t* tests (*p* < .05; fold change, >1.25) to identify miRNAs highly expressed among the three groups (Nakaya et al., 2011). The Shapiro–Wilk normality test assessed the normal distribution of the data. Power analysis indicated how many samples would be required per group to validate the results of the microarray considering the confidence level of 0.95 and target *p*‐value of .05.

## 3 RESULTS

### 3.1 Data sample and experimental design

No significant differences were found in the anthropometric and clinical characteristics (Appendix S1). Only the plasma glucose measurements used for the diagnosis were different among the three groups (Appendix S1).

**FIGURE 1:**
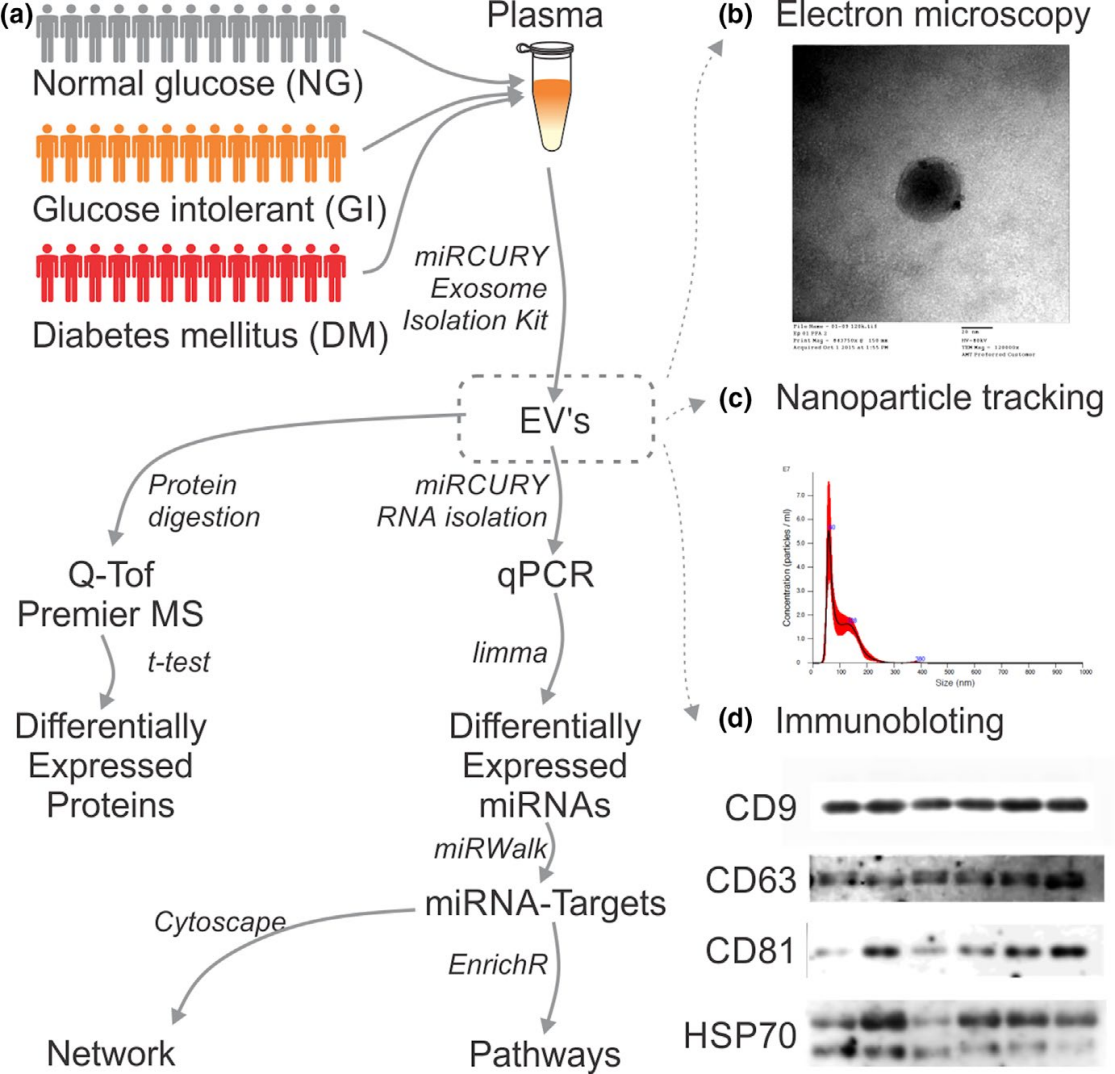
Workflow of the experimental design. Plasma from normal glucose (NG; *n* = 22), glucose intolerant (GI; *n* = 20), and newly diagnosed diabetes (DM; *n* = 20) subjects were randomly collected from the ELSA‐Brazil Study based on the results of fasting glycemia, glycemia after the oral glucose tolerance test, and glycated hemoglobin. Microvesicles were isolated following the manufacturer's instructions, and total RNA was isolated and used for miRNA microarray analysis. The proteins present in the plasma‐derived microvesicles were isolated and evaluated by mass spectrometry. Extracellular vesicles were characterized by (b) transmission electron microscopy, (c) particle and size distribution using nanoparticle tracking analysis, and (d) immunoblotting for anti‐CD9, anti‐CD81, and anti‐HSP70

EVs were isolated from human plasma and analyzed for size by transmission electron microscopy, nanoparticle tracking analysis (NTA) and western blotting (Figure 1b–d; Appendix S1). The plasma‐derived EVs were within the normal range for size (30–150 nm in diameter) and presented a circular shape, consistent with previously reported EV characteristics (Simpson et al., 2009; Théry et al., 2001).

Although EVs have lipid, protein, and RNA components, the overall EV composition is quite different from those of parental cells, suggesting an active sorting mechanism after EV secretion. MicroRNAs and proteins derived from isolated EVs of NG, GI, and DM subjects were analyzed using microarray and mass spectrometry, respectively, as depicted in the workflow of the experimental design (Figure 1).

### 3.2 Plasma extracellular vesicle proteins and serum ferritin levels

Forty‐eight proteins were identified by mass spectrometry in plasma EVs obtained from the three groups (Appendix S1). Our results revealed a high percentage of proteins linked to Gene Ontology (GO) terms (Choi et al., 2013, 2015; Kim et al., 2013; Simons & Raposo, 2009; Zhang et al., 2019) such as *cytosol*, *cytoplasmic*, and *vesicle*, suggesting that the proteins were likely derived from plasma EVs and not contaminants. GO analysis was conducted to determine the functions of plasma‐EV‐associated proteins. The proteins were sorted into categories according to their ontology as determined from their GO annotation terms (data not shown). The annotated biologic processes of the proteins revealed enrichment of plasma EV‐associated proteins related to *transport*, *immune response*, *energy pathways*, *cell growth and or/maintenance*, and *protein metabolism*.

**FIGURE 2:**
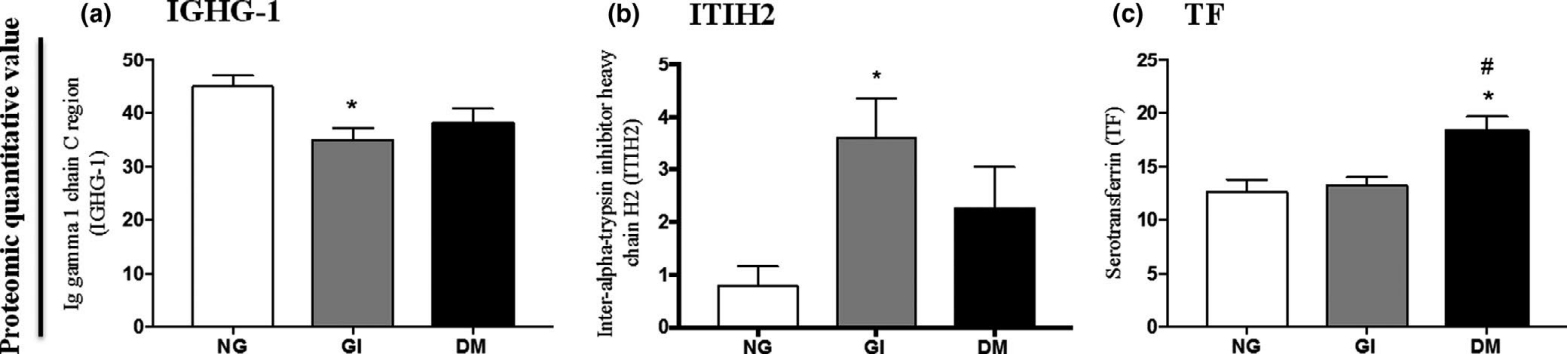
Multiple variance analyses of immunoglobulin heavy constant gamma 1 (IGHG‐1) (a), interalpha‐trypsin inhibitor heavy chain H2 (ITIH2) (b) and serotransferrin (TF) protein (c) quantified by mass spectrometry in plasma microvesicles. NG, normal glucose (*n* = 7); GI, glucose intolerant (*n* = 7); DM, newly diagnosed diabetes mellitus (*n* = 7). The data were presented as means ± SEM and were analyzed by one‐way ANOVA and Tukey's post hoc test. *p < .05 compared with NG; **p < .01 compared with NG; #*p* < .05 compared with GI

**FIGURE 3:**
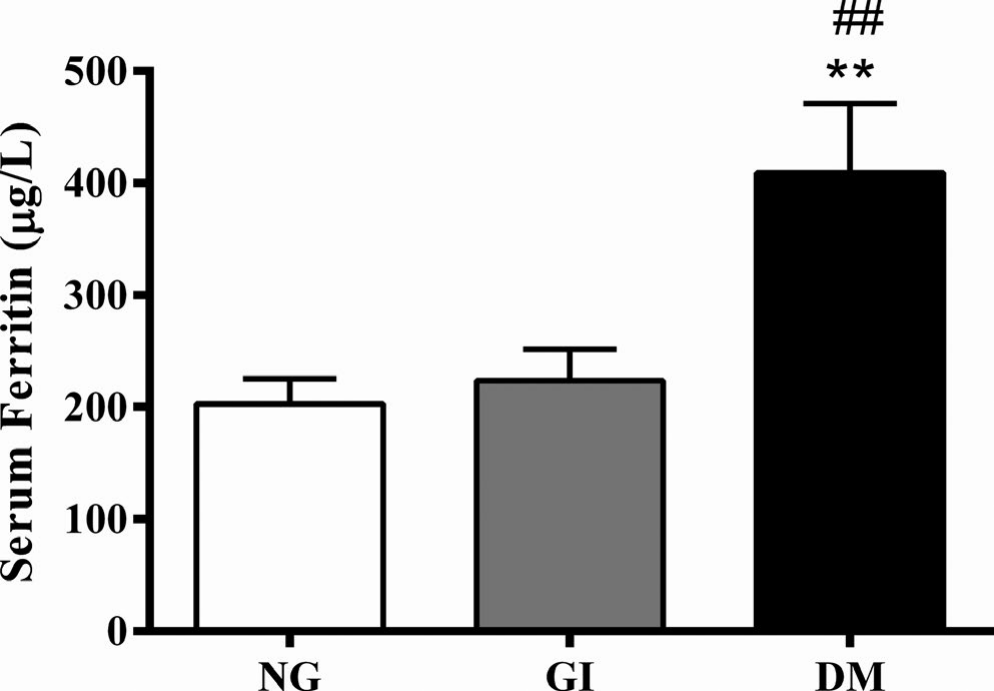
Serum ferritin levels. NG: normal glucose; GI: glucose intolerant; DM: newly diagnosed diabetes. *N* = 20–22 per group. The data were presented as means ± SEM and were analyzed by one‐way ANOVA and Tukey's post hoc test. **p < .01 compared with NG; ##*p* < .01 compared with GI

We also compared the 48 proteins identified in all groups with those reported in previous EV studies included in the EVpedia database (Choi et al., 2013, 2015; Kim et al., 2013; Simons & Raposo, 2009; Zhang et al., 2019). Thirty‐nine proteins (81.25%) overlapped among the 245 proteins in EVpedia‐loaded human plasma extracellular vesicles (human, plasma, normal, #274870810101), indicating an essential profile of the plasma‐EV proteome. We cannot exclude the possible interference of contaminants in this analysis as detected by the presence of albumin and apolipoprotein B contamination in all samples analyzed. Three proteins identified in the EV proteome were within the 39 proteins that overlapped with the database and were differentially expressed among the groups. The immunoglobulin heavy constant gamma 1 (IGHG‐1) was significantly decreased (22%) in the plasma EVs of GI subjects compared with that in the NG group (Figure 2a). The EVs from the GI group also showed a significant increase (4.6‐fold) in the interalpha‐trypsin inhibitor heavy chain H2 (ITIH2) protein compared with that in the NG group (Figure 2b). The serotransferrin protein (TF) level was increased in subjects with diabetes compared with that in the NG and GI groups (45% and 38%, respectively; Figure 2c). Additionally, the serum ferritin levels were increased in the DM group by 102% and 83% compared with those in the NG and GI groups, respectively (Figure 3). The association between serum ferritin levels and diabetes are not completely understood, but it has been found a high prevalence of excess iron in patients with metabolic syndrome (Bozzini et al., 2005).

### 3.3 Plasma extracellular vesicles miRNA

**FIGURE 4:**
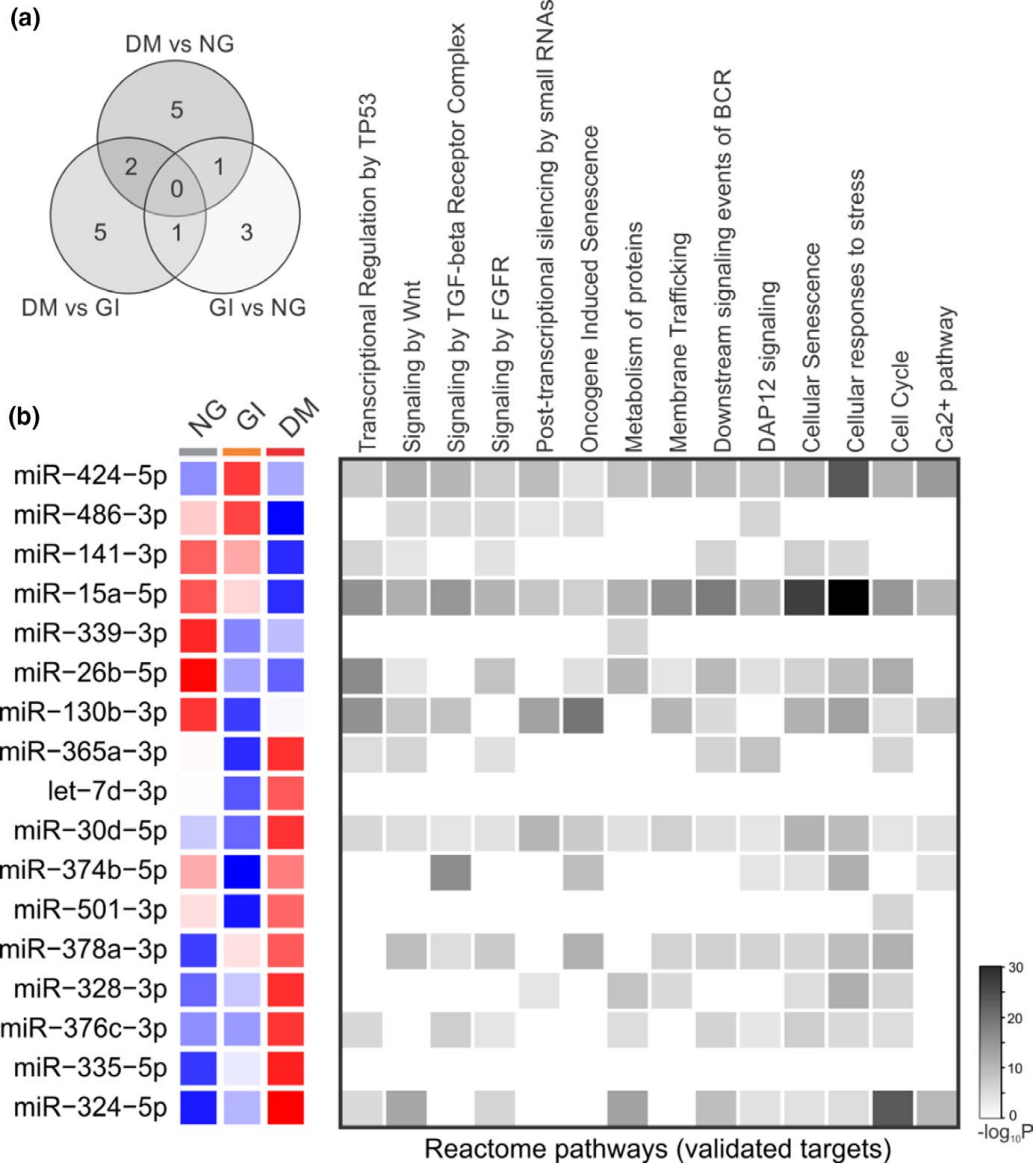
Profiling plasma‐derived microvesicle microRNAs in the three groups. (a) The Venn diagram indicates the number of differentially expressed microRNAs in each pairwise comparison. (b) Heat‐map with the fold change over the normal glucose group for the 17 miRNAs expressed in all groups. The color scale illustrates the row‐wise *z*‐score expression by microRNA; red and blue represent over and under mean expression, respectively. Each square represents the group means. The left square represents differentially expressed microRNAs enriched in the Reactome pathway target. NG, normal glucose (*n* = 15); GI, glucose intolerant (*n* = 15); DM, newly diagnosed diabetes mellitus (*n* = 15)

Plasma‐derived EV miRNA candidates associated with newly diagnosed DM were assessed using miRNA microarrays. As represented in the scatter plot (Figure 4b), seventeen miRNAs were differentially expressed (DEMiRs) in the plasma EVs of the three groups. Pairwise comparison of the results was then performed (Appendix S1), and eight DEMiRs were found to be different between the DM and NG groups. Three of these miRNAs were upregulated (miR‐141‐3p, miR‐15a‐5p, and miR‐26b‐5p) and five were downregulated (miR‐378a‐3p, miR‐328‐3p, miR‐376c‐3p, miR‐335‐5p, and miR‐324‐5p) in DM plasma‐derived EV samples compared with those in the NG group (*p* < .05; Appendix S1). Pairwise comparison between the DM and GI groups identified nine DEMiRs: two were upregulated (miR‐141‐3p and miR‐486‐3p) and seven were downregulated (miR‐365a‐3p, let‐7d‐3p, miR‐30d‐5p, miR‐374b‐5p, miR‐501‐3p, miR‐376c‐3p, and miR‐324‐5p) (*p* < .05; Appendix S1). The comparison between the GI and NG groups identified five DEMiRs: one downregulated (miR‐424‐5p) and four upregulated (miR‐339‐3p, miR‐26b‐5p, miR‐130b‐3p, and miR‐374b‐5p) (*p* < .05; Appendix S1) (Figure 4b).

The number of DEMiRs between the circles in Figure 4a indicates the pairwise comparison among the groups, exhibiting the following patterns: (a) miR‐141‐3p was upregulated, and miR‐324‐5p and miR‐376c‐3p were downregulated in both comparison of DM vs NG or vs GI; (b) miR‐26b‐5p was upregulated in the DM and GI groups compared with that in the NG group, as indicated in the Venn diagram (Figure 4a); and (c) The expression of miR‐374b‐5p was downregulated in the DM group compared with that in the GI group and upregulated in the GI group compared with that in the NG group (Figure 4a).

Three of the seventeen DEMiRs were correlated with all reactome pathways identified in Figure 4b: miR‐424‐5p, miR‐15a‐5p, and miR‐30d‐5p. Considering the seventeen DEMiRs identified in the microarray analyses, 59% of them were correlated with the following signaling pathways: Wnt (Wingless Int‐1), TGF‐β (transforming growth factor‐beta) receptor complex, FGFR (fibroblast growth factor receptor), downstream events of BCR (B‐cell receptor), and DAP12 (DNAX activation protein of 12 kDa). Additionally, 65% of the DEMiRs were correlated with cellular senescence, the cellular response to stress, and the cell cycle (Figure 4b).

**FIGURE 5:**
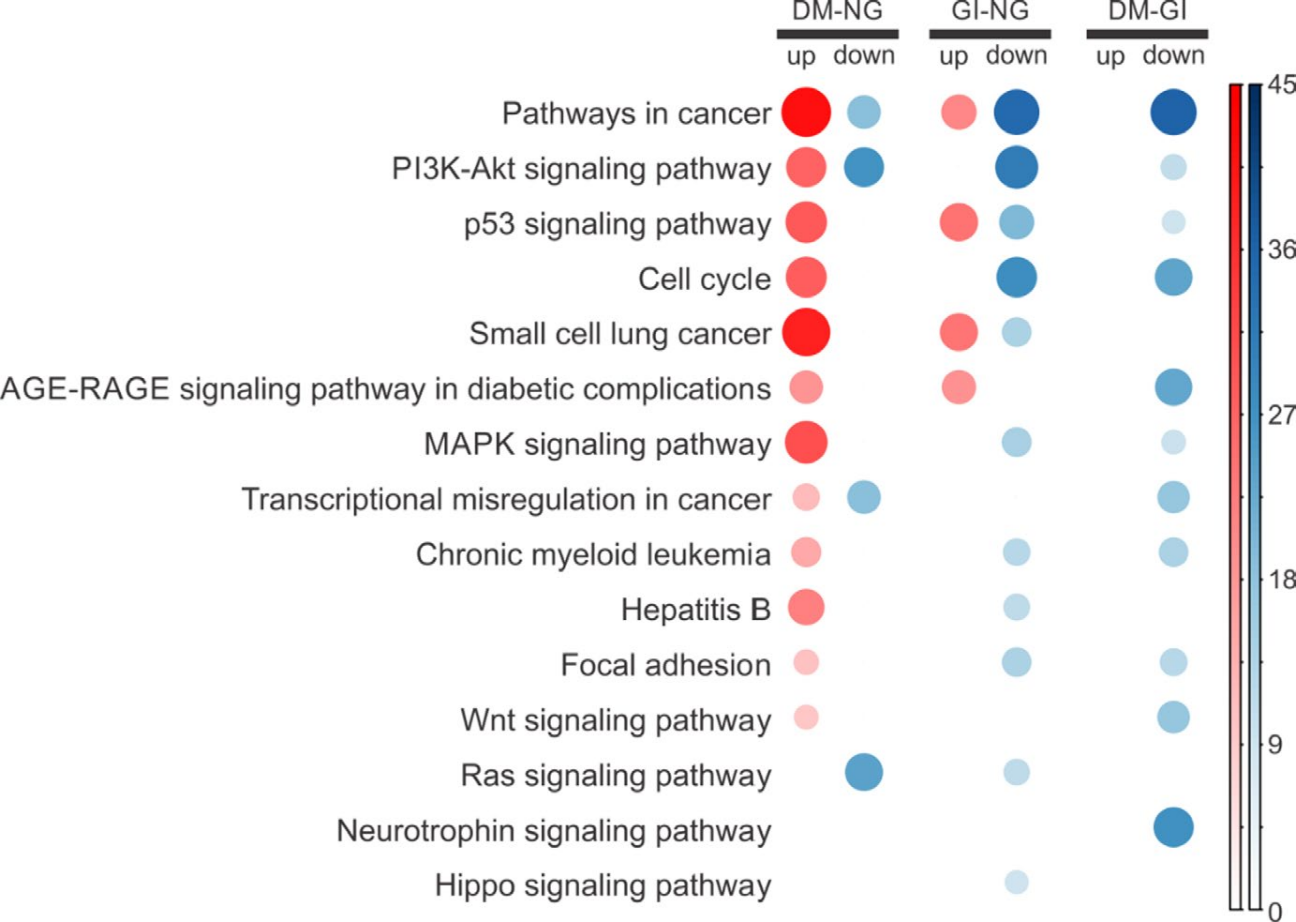
Findings of gene set enrichment analysis. Gene expression Corrplot analysis represents blood transcription modules and functional pathways that were increased (red) or reduced (blue) in the peripheral blood of each group. DM, newly diagnosed diabetes mellitus (*n* = 15); NG, normal glucose (*n* = 15); GI, glucose intolerant (*n* = 15); AGE, advanced glycation end‐products; RAGE, receptor for advanced glycation end‐products; MAPK, mitogen‐activated protein kinase

To describe the significantly different functional content of DEMiRs in the pairwise comparison among the three groups (DM vs NG; DM vs GI; GI vs NG; Figure 4a), we performed enrichment analysis (Figure 5). The progression of diabetes was mainly associated with dysregulated cancer pathways, PI3 K‐Akt signaling, p53 signaling, and the cell cycle (Figure 5).

**FIGURE 6:**
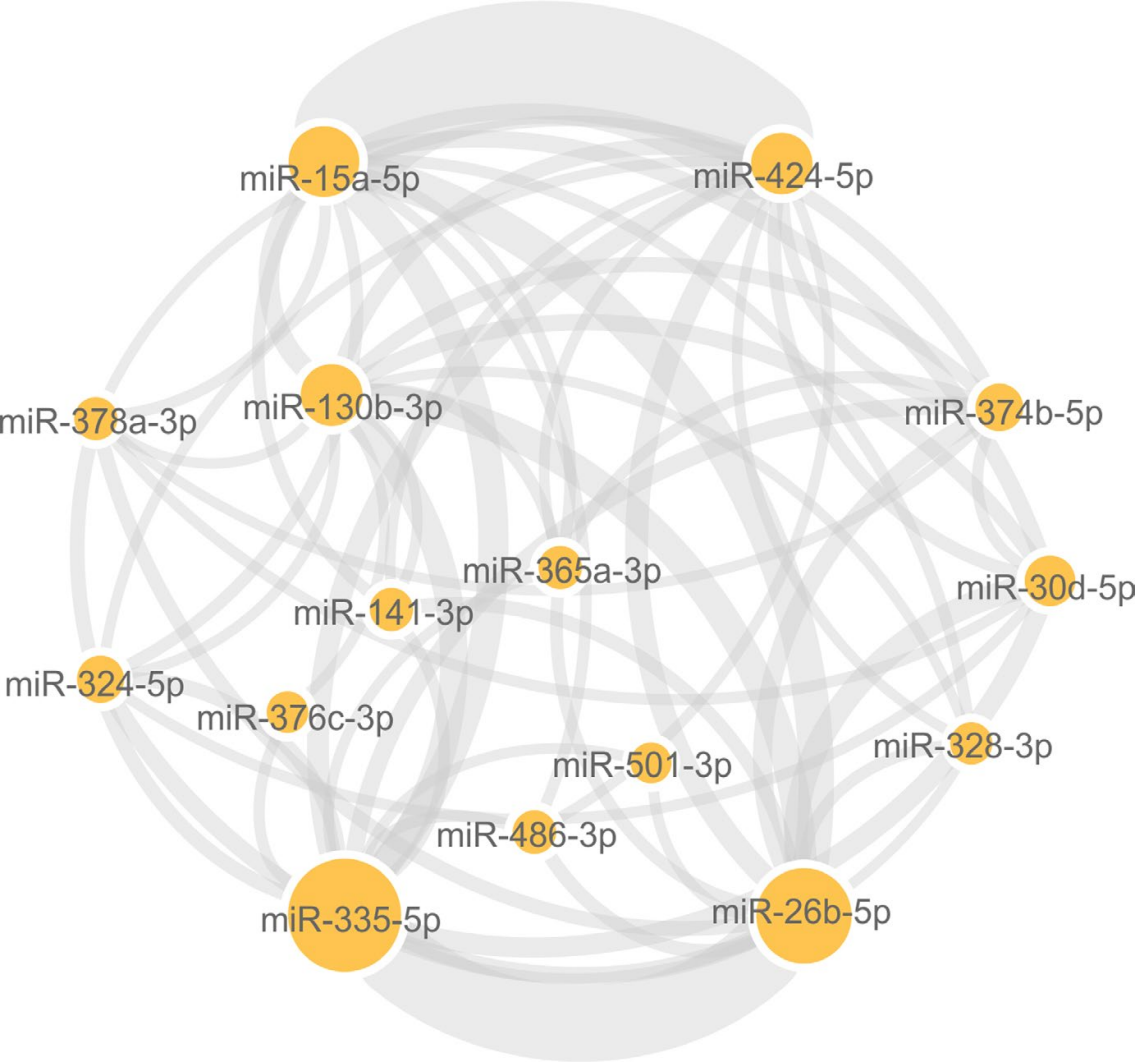
Regulatory network of diabetes‐associated genes and their target miRNAs. Cytoscape software was used to analyze a list containing 17 differentially expressed miRNAs capable of separating subjects with newly diagnosed diabetes mellitus from those with glucose intolerance and normal glucose based on their respective fold change and *p* values. Circle nodes represent differentially expressed miRNAs. A minimum of 10 targets forms the lines connecting the miRNAs, and the thickness and the size of the nodes are directly proportional to the number of targets. miR, microRNA

Cytoscape software revealed that common targets connected 15 miRNAs of all the DEMiRs herein described. The regulatory network of miRNAs linked by common targets is presented in Figure 6. miR‐15a‐5p, miR‐424‐5p, miR‐335‐5p, and miR‐26b‐5p shared the highest number of target genes in the regulatory network (Figure 6).

## 4 DISCUSSION

EVs are highly enriched in proteins with various functions such as those involved in cell penetration, invasion, fusion, the stress response, EV release, membrane transport, and biogenesis (McHeyzer‐Williams et al., 2012; Vlassov et al., 2012). Although individual variation was found in mass spectrometry analysis in the present study, some of the shared proteins among all the plasma EV samples suggested the presence of a typical proteome among the three groups. It is worth considering that EV isolation from serum or plasma is especially difficult due to the small volume available, its high viscosity, protein concentration, and the presence of other particles, mainly lipoproteins (Brennan et al., 2020). Lipoprotein contamination is a problem with most of the commercial kits used to isolate EVs (Macías et al., 2019), and we also found impurities, as detected by the presence of albumin and apolipoprotein B contamination in all samples analyzed by gas chromatography (Appendix S1). Next, we cannot exclude the possible interference of contaminants in this analysis. To our knowledge, no published study exists regarding the proteomics of human plasma EVs related to diabetes onset in middle‐aged men.

Immunoglobulins, such as IGHG‐1, which were significantly decreased in the plasma EVs of GI subjects, are membrane‐bound or secreted glycoproteins produced by B lymphocytes. In the recognition phase of humoral immunity, the membrane‐bound immunoglobulins serve as receptors that, upon binding to a specific antigen, trigger the clonal expansion and differentiation of B‐lymphocytes into immunoglobulin‐secreting plasma cells (Calvet & Yoshikawa, 2001; Schroeder & Cavacini, 2010). DM patients have increased rates of infections, partially explained by a decreased T and B cell‐mediated immune response (Pozzilli & Leslie, 1994; Shah & Hux, 2003). The significant reduction of EV IGHG‐1 in the GI group compared with that in the NG group suggests the low immune response in these subjects. Likewise, EVs from the GI group also presented a higher content of interalpha‐trypsin inhibitor heavy chain H2 (ITIH2) protein. ITIH2 is a member of a protein family structurally associated with plasma serine protease inhibitors involved in extracellular matrix stabilization according to GeneCards. Together with three other proteins (APO4, C7, and CLU), ITIH2 is considered a useful biomarker to detect the early stages of diabetic retinopathy, a common microvascular DM complication (Garcia‐Ramirez et al., 2007; Jin et al., 2016). The plasma EV proteomic analysis presented herein highlights how significant differences can be observed in patients in the glucose intolerance phase compared with those with a normal glucose status.

Furthermore, the serum ferritin level was found to be increased in the DM group analyzed in this study. Serum ferritin levels reflect iron storage, and a positive association among the serum ferritin and fasting plasma glucose levels, glycated hemoglobin, and insulin resistance has been reported (Chen et al., 2017, 2018). Iron overload might be one of the causes of metabolic syndrome, as insulin sensitivity improves when the ferritin level decreases (Fernández‐Real et al., 2002). In 1998, the serum ferritin concentration was proposed to be a component of metabolic syndrome (Fernández‐Real et al., 1998, 2002). In fact, there is a high prevalence of excess iron in patients with metabolic syndrome (Bozzini et al., 2005). Iron status at both extremes is associated with premature death and has clinical importance for diagnosing patients with DM at the proper time.

However, we found that the serotransferrin protein levels were increased only in plasma EVs from middle‐aged men with diabetes. Serotransferrin delivers iron to all cells (Gkouvatsos et al., 2012) and has increased turnover in type 2 diabetes with enhanced systemic oxidative stress (Golizeh et al., 2017). Iron stores are associated with an enhanced risk of developing type 2 diabetes (Fernández‐Real et al., 2015). The liver is the major iron reservoir, and the increased serotransferrin EV protein in DM patients suggests that this is the first organ to start the body‐talk‐reaction through EVs in the disease onset.

After analyzing the EV protein content, we investigated the microRNA expression in EVs in individuals with glucose metabolism disturbance comparing the GI and DM groups with the NG group. Among the seventeen DEMiRs identified in the samples analyzed in this study, miR‐26b‐5p expression was upregulated in both the DM and GI groups. Stępień et al. (2018) described significantly increased expression of miR‐26b‐5p, which targets anti‐angiogenic genes, in plasma ectosomes from type‐2 diabetes patients. Ectosomes are also extracellular vesicles that retain many features of their parental cells, and the primary blood sources are platelets and endothelial cells (Choi et al., 2015).

At diabetes onset, DM patients showed upregulation of miR‐141‐3p and downregulation of miR‐324‐5p and ‐376c‐3p. miR‐141‐3p is associated with mitochondria functions and is a potential biomarker of various diseases, including hepatic dysfunction in obesity (Ji et al., 2015), primary biliary cirrhosis (Tan et al., 2014), bladder cancer (Canturk et al., 2014), and colorectal cancer (Della Vittoria Scarpati et al., 2014). MiR‐141‐3p also modulates the mRNA expression of transferrin receptor via iron‐regulatory protein interplay in two human cell lines, human erythroleukemia K562 cells that have been extensively used for transferrin receptor studies and human colon adenocarcinoma SW480 cells (Miyazawa et al., 2018). MiR‐324‐5p was proven to be involved in many human diseases by modulating its downstream targets, such as mitochondrial fission regulator 1 (Mtfr1) in cardiomyocytes (Wang et al., 2015) and the NF‐κB signaling pathway that regulates fibrosis and inflammation (Song et al., 2015). Downregulation of miR‐376c‐3p was reported in plasma EVs from patients with paroxysmal nocturnal hemoglobinuria (PNH) (Teruel‐Montoya et al., 2019). PNH is a rare systemic disease associated with the deficiency of certain proteins in the erythrocyte membrane that causes intravascular hemolysis, the production of inflammatory mediators and systemic release of hemoglobin (Brodsky, 2014). Both PNH and DM are associated with red blood cell function impairments (Tomaiuolo, 2014).

Finally and interestingly, the expression of miR‐374b‐5p was downregulated in the DM groups compared with that in the GI group and was upregulated in the GI group compared with that in the NG group. The dysregulation of miR‐374b‐5p has been implicated in several disorders, including obesity, calcific aortic stenosis, and ischemic stroke (Jones et al., 2017; Sun et al., 2018; Tan et al., 2017). Patients with IgA nephropathy (IgAN), the most common glomerulonephritis worldwide, exhibit higher miR‐374b expression in B cells compared with controls, a finding that is associated with B‐cell proliferation and aberrant IgA1 glycosylation (Hu et al., 2015).

The results described herein are highly relevant for developing clinically applicable diagnostic/prognostic biomarkers in diabetes and might be expanded. However, some limitations must be considered in this study, such as the homogeneity of the analysis performed only in middle‐aged men, hemolysis analysis performed by visual inspection and delta Cq (miR‐23a – miR‐451, with mean and standard deviation of 5.7 ± 1.7, Blondal et al., 2013), the small number of samples and the lack of validation of the results using other different techniques.

We report for the first time the proteomic and miRNA signatures in plasma EVs isolated from glucose‐intolerant and newly diagnosed subjects with diabetes. We identified a potential multiple‐EV biomarker panel of five DEMiRs (miR‐141‐3p, ‐324‐5p, 376c‐3p, ‐26b‐5p, and ‐374b‐5p) and 3 proteins (IGHG‐1, ITIH2, and TF), which have potential diagnostic/prognostic importance for long‐term diabetic complications. Previous studies have suggested the potential usefulness of circulating EVs as biomarkers in diabetes (Beuzelin & Kaeffer, 2018; Guay & Regazzi, 2017; Sáez et al., 2019). However, to our knowledge, this is the first report on the quantitative assessment of plasma EV proteins and their miRNA cargos prior to disease onset. We reported that plasma‐derived EV proteins and miRNA cargos might be used as diagnostic/prognostic biomarkers for diabetic complications. However, complementary studies including in vitro analysis are required to fully understand and confirm the involvement of these potential proteins and miRNAs in diabetes development.

## STUDY APPROVAL

The ELSA‐Brasil protocol was approved at University of Sao Paulo (USP) by the institutional review boards addressing research in human participants. All the participants signed a written informed consent form. All the protocols were carried out in accordance with the American Diabetes Association guidelines (Association, 2018).

## CONFLICT OF INTEREST

The authors have declared that no conflict of interest exists.

## AUTHOR CONTRIBUTIONS

Design of the study: L.N.M., P.A.L., A.C.R., R.C., S.M.H. Collection of the samples: L.N.M., P.A.L., T.D.A.S., A.A.B., M.E.G.S., T.C.A.L., M.H.H., S.L., I.M.B. Laboratory measurements: L.M.G.F. Transmission electron microscopy analysis: D.P.C. NanoSight analysis: F.T.B. Statistical analysis: F.M.F., H.I.N. Interpretation of the findings and writing of the manuscript: L.N.M, P.A.L., F.M.F., A.C.R., T.S.S, R.G., T.C.P.‐C., H.I.N., R.C., S.M.H. All authors have approved the final manuscript version for publication and have accepted accountability for all aspects of the work and for authorship.

## Supporting information



AppendixS1
