# Supplementary material for: Profiling plasma‐extracellular vesicle proteins and microRNAs in diabetes onset in middle‐aged male participants in the ELSA‐Brasil study
Source: Physiol Rep. 2021 Feb 15;9(3):e14731. doi: 10.14814/phy2.14731 (PMC7883809; doi:10.14814/phy2.14731)

Profiling plasma-derived extracellular vesicle proteins and microRNAs in diabetes onset in middle-aged male participants in the Brazilian Longitudinal Study of Adult Health

Laureane N Masi[1*], Paulo A Lotufo[2], Frederico M Ferreira[3], Alice C Rodrigues[4], Tamires D A Serdan[1], Talita Souza-Siqueira[1], Aécio A Braga[5], Magda E G Saldarriaga[5], Tatiana C Alba-Loureiro[1], Fernanda T Borges[1], Diego Cury[6], Mario H Hirata[5], Renata Gorjão[1], Tania C Pithon-Curi[1], Simão A Lottenberg[7], Ligia M G Fedeli[2], Helder I Nakaya[4], Isabela M Bensenor[2], Rui Curi[1], Sandro M Hirabara[1]

[1]Interdisciplinary Post-graduate Program in Health Sciences, Cruzeiro do Sul University, Sao Paulo, Brazil.
[2]Center for Clinical and Epidemiologic Research, University of Sao Paulo, Brazil.
[3] Department of Pathology, University of Sao Paulo, School of Medicine, Sao Paulo, Brazil.
[4]Department of Pharmacology, Institute of Biomedical Sciences, University of Sao Paulo, Sao Paulo, Brazil.
[5]Faculty of Pharmaceutical Sciences, University of São Paulo, Sao Paulo, Brazil.
[6]Department of Anatomy, Institute of Biomedical Sciences, University of Sao Paulo, Sao Paulo, Brazil.
[7]Hospital das Clinicas, Faculty of Medicine, University of Sao Paulo, Sao Paulo, Brazil.

*Corresponding author: Cruzeiro do Sul University. Rua Galvao Bueno, 868, Liberdade, São Paulo, SP, CEP 01506-000 (Brasil) Tel. +55-11-3385-3103.
E-mail: laureane.masi@cruzeirodosul.edu.br

Supplementary materials

Suppl. 1. Anthropometric and clinical data of the participants.

Data	NG	GI	DM
Male (N)	22	20	20
Age (years)	43.3 ± 0.9	46.1 ± 0.7	44.5 ± 0.6
Body mass (kg)	80.7 ± 1.7	82.8 ± 2.1	82.2 ± 1.5
Height (cm)	170.4 ± 1.6	172.2 ± 1.8	172.4 ± 1.6
BMI (kg/m^2)	27.6 ± 0.3	27.7 ± 0.3	27.4 ± 0.3
Waist circunference (cm)	91.4 ± 1.2	94.5 ± 1.1	94.5 ± 1.4
Waist / hip ratio (WHR)	0.9 ± 0.01	0.9 ± 0.01	0.9 ± 0.01
Plasma glucose (mg/dL)	**103.1 ± 1.1**	**107.8 ± 1.6**	**121.1 ± 3.7[a,b]**
Insulinemia (mcUI/mL)	7.4 ± 1.2	7.2 ± 0.8	10.2 ± 1.3
Glycated hemoglobin (%)	**5.1 ± 0.1**	**5.2 ± 0.1**	**6.1 ± 0.3[a,b]**
Plasma glucose pos-overload (mg/dL)	**113.6 ± 2.9**	**157.2 ± 2.8[a]**	**170.1 ± 12.5[a]**
Insulinemia pos-overload (mcUI/mL)	50.3 ± 7.8	96.4 ± 28.1	70.7 ± 12.9
HOMA-IR	1.9 ± 0.3	2.0 ± 0.2	3.0 ± 0.4
Triglycerides (mg/dL)	117.6 ± 11.2	131.4 ± 8.0	134.4 ± 9.0
Total cholesterol (mg/dL)	216.2 ± 7.0	215.4 ± 9.6	204.9 ± 6.1
HDL-cholesterol (mg/dL)	51.8 ± 2.4	47.4 ± 2.1	50.7 ± 2.2
LDL-cholesterol (mg/dL)	141.0 ± 6.9	141.7 ± 8.1	127.3 ± 6.1
Gama glutamil transferase (U/L)	43.9 ± 7.9	34.1 ± 3.2	38.1 ± 3.2
TGO/AST (U/L)	25.0 ± 1.2	26.3 ± 1.0	29.7 ± 2.9
TGP/ALT (U/L)	31.6 ± 2.4	33.2 ± 2.1	43.7 ± 5.3
Serum Uric acid (mg/dL)	6.1 ± 0.3	6.9 ± 0.2	6.9 ± 0.2
C-Reactive protein (mg/L)	1.8 ± 0.3	1.8 ± 0.3	1.6 ± 0.3

Data presented as average ± standard error of mean (SEM); (a) $p < 0.05$ *vs* NG; (b) $p < 0.05$ *vs* GI, one-way ANOVA with Tukey post-test. NG = normoglycemia; GI = glucose intolerant; DM = newly diagnosed diabetes; BMI = Body mass index (kg/m^2); HOMA-IR = insulin resistance index; TGO/AST = aspartate aminotransferase; TGP/ALT = alanine aminotransferase. Parameters with significant differences are indicated in bold.

Suppl. 2. Individual clinical data used as criteria for T2DM diagnosis.

	NG			GI			DM		
**Participant**	**Glycaemia (mg/dL) < 100**	**Glycaemia OGTT (mg/dL) < 140**	**HbA1c % < 5,7**	**Glycaemia (mg/dL) >100 <126**	**Glycaemia OGTT (mg/dL) >140 <200**	**HbA1c % >5,7 <6,4%**	**Glycaemia (mg/dL) > 126**	**Glycaemia OGTT (mg/dL) > 200**	**HbA1c % > 6,5%**
**1**	103	122	5	110	141	5	124	194	6
**2**	104	120	5	115	154	5	112	**203**	5
**3**	98	133	5	92	146	5	**163**	**244**	**7**
**4**	103	132	6	106	153	6	**150**		6
**5**	109	113	5	112	160	6	114	103	**7**
**6**	101	108	5	105	172	5	106	**212**	4
**7**	101	91	5	110	177	5	114	118	**7**
**8**	101	93	5	111	189	5	103	104	**7**
**9**	107	119	5	107	160		107	131	**7**
**10**	111	122	5	104	159	6	117	173	**7**
**11**	96	84	5	101	150	6	104	180	**7**
**12**	103	125	5	116	151	5	**133**	173	**7**
**13**	103	106	6	117	143	6	109	156	**8**
**14**	116	123	5	93	145	5	118	155	**8**
**15**	96	108	5	113	162	5	115	94	**8**
**16**	108	106	5	113	155	5	**136**	**297**	**7**
**17**	100	114	5	105	165	4	100	**229**	**8**
**18**	100	115	5	102	143	5	**137**	150	**7**
**19**	100	98	5	119	171	5	**132**	**213**	**9**
**20**	102	127	6	105	147	5	**127**	113	**7**
**21**	109	133	5						
**22**	98	107	5						

Suppl. 3. The plasma proteins precipitated by miRCURY Exosome Isolation Kit of NG, GI and DM subjects identified by mass- spectrometry.

Identified Proteins (48)	UniProt	NG (n = 7)	GI (n = 7)	DM (n = 7)
Serum albumin	H7C013	130.77 ± 7.28	122.67 ± 5.27	118.89 ± 5.02
Alpha-2-macroglobulin	P01023	76.63 ± 6.25	66.38 ± 3.18	72.79 ± 4.79
Complement C3	P01024	54.98 ± 6.11	61.54 ± 3.21	56.57 ± 8.04
Ig kappa chain C region	P01834	36.27 ± 2.02	34.84 ± 2.26	37.95 ± 2.41
**Ig gamma-1 chain C region**	**P01857**	**45.01 ± 2.04**	**35.04 ± 2.18***	**37.98 ±2.89**
Ig lambda-2 chain C regions (Fragment)	P0CG05	29.08 ± 1.66	28.60 ± 1.71	28.41 ± 1.91
Apolipoprotein B-100	P04114	15.90 ± 3.74	22.13 ± 2.96	22.36 ± 5.47
Ig alpha-1 chain C region	P01876	17.94 ± 3.04	20.03 ± 2.80	19.32 ± 2.85
Ig gamma-3 chain C region	P01860	32.78 ± 2.93	35.66 ± 2.33	33.97 ± 2.03
Complement C4-B	P0C0L5	16.08 ± 2.88	16.16 ± 2.57	17.11 ± 4.09
Ig mu chain C region	P01871	16.40 ± 1.94	19.37 ± 3.15	15.02 ± 1.67
**Serotransferrin**	**P02787**	**12.63 ± 1.15**	**13.24 ± 0.81**	**18.31 ± 1.39*#**
Ig gamma-2 chain C region (Fragment)	P01859	27.26 ± 3.05	25.73 ± 1.99	22.73 ± 1.98
Fibrinogen alpha chain	P02671	7.94 ± 2.63	12.32 ± 3.00	13.11 ± 2.61
Fibrinogen gamma chain	P02679	11.87 ± 3.21	11.31 ± 1.70	12.07 ± 2.57
Apolipoprotein A-I	P02647	10.25 ± 1.26	11.08 ± 0.87	13.07 ± 1.70
Fibrinogen beta chain	P02675	7.92 ± 2.75	11.56 ± 3.31	10.25 ± 2.05
Ig gamma-4 chain C region (Fragment)	P01861	26.66 ± 2.02	25.98 ± 0.95	26.71 ± 3.23
Ceruloplasmin	P00450	4.55 ± 1.37	6.7 ± 1.22	5.46 ± 1.12
Haptoglobin	P00738	6.79 ± 1.67	3.76 ± 1.51	5.29 ± 1.37
Complement factor H	P08603	3.91 ± 1.31	3.59 ± 0.92	4.38 ± 1.13
Ig kappa chain V-III region SIE	P01620	4.08 ± 0.82	3.53 ± 0.59	3.99 ± 0.29
Ig lambda chain V-III region LOI	P80748	4.57 ± 0.95	3.30 ± 0.49	3.48 ± 0.72
C4b-binding protein alpha chain	P04003	3.01 ± 0.82	2.97 ± 0.83	3.31 ± 0.86
Immunoglobulin lambda-like polypeptide 5	B9A064	27.32 ± 3.05	23.57 ± 1.22	22.42 ± 1.41
Alpha-1-antitrypsin	P01009	2.97 ± 0.34	2.54 ± 0.65	3.18 ± 0.86
**Inter-alpha-trypsin inhibitor heavy chain H2**	**P19823**	**0.79 ± 0.37**	**3.60 ± 0.75***	**2.26 ± 0.78**
Hemoglobin subunit beta	P68871	0.79 ± 0.31	2.36 ± 1.23	2.85 ± 1.24
Apolipoprotein E	P02649	1.32 ± 0.58	2.01 ± 0.66	2.64 ± 0.71
Ig kappa chain V-IV region Len	P01625	2.72 ± 0.66	1.72 ± 0.49	1.93 ± 0.43
Fibronectin	P02751	1.15 ± 0.90	1.77 ± 0.61	1.85 ± 0.57
Plasminogen	P00747	1.03 ± 0.52	2.06 ± 0.64	1.45 ± 0.38
Uncharacterized protein (Fragment)	H0Y858	1.51 ± 0.34	1.72 ± 0.28	1.74 ± 0.54
Protein IGKV2D-28	A0A075B6P5	1.62 ± 0.37	1.39 ± 0.46	1.75 ± 0.57
Inter-alpha-trypsin inhibitor heavy chain H1	P19827	1.59 ± 0.56	1.60 ± 0.46	1.19 ± 0.50
CD5 antigen-like	O43866	0.85 ± 0.44	1.86 ± 0.74	0.92 ± 0.40
Immunoglobulin J chain (Fragment)	P01591	0.77 ± 0.36	1.49 ± 0.52	1.55 ± 0.46
Ig kappa chain V-I region EU	P01598	1.81 ± 0.69	1.02 ± 0.41	1.43 ± 0.47
Protein AMBP	P02760	1.30 ± 0.34	1.48 ± 0.26	0.69 ± 0.18
Apolipoprotein A-II	P02652	1.11 ± 0.39	1.29 ± 0.43	0.96 ± 0.43
Apolipoprotein C-III	P02656	1.09 ± 0.45	0.78 ± 0.24	1.06 ± 0.36
Hemopexin	P02790	0.78 ± 0.37	1.69 ± 0.73	0.39 ± 0.19
Hemoglobin subunit alpha	P69905	0.33 ± 0.22	1.18 ± 0.59	1.19 ± 0.61
Glucose-6-phosphate 1-dehydrogenase	P11413	0.00 ± 0.00	1.65 ± 1.65	0.00 ± 0.00
Apolipoprotein C-II isoform 1	P02655	0.26 ± 0.26	0.51 ± 0.26	0.25 ± 0.16
Protein IGHV5-51 (Fragment)	A0A0C4DH38	0.53 ± 0.39	0.39 ± 0.18	0.00 ± 0.00
Vitamin D-binding protein	P02774	0.33 ± 0.22	0.00 ± 0.00	0.50 ± 0.38
Apolipoprotein(a)	P08519	0.00 ± 0.00	0.13 ± 0.13	0.50 ± 0.50

Suppl. 4. Analysis of the miRNAs from plasma extracellular vesicles from NG, GI, and DM subjects, evaluated by micro-array and normalized by the CT global mean.

miRNA	DM x NG (FC)	P value	DM x GI (FC)	P value	GI x NG (FC)	P value
hsa-let-7a-5p	1.0968	0.5171	1.0471	0.7465	1.0475	0.7442
hsa-let-7b-3p	-1.4898	0.2415	-1.0037	0.9909	-1.4843	0.2458
hsa-let-7b-5p	1.3261	0.0898	1.1206	0.4852	1.1834	0.3041
hsa-let-7c-5p	1.0379	0.8446	1.1620	0.4312	-1.1196	0.5528
**hsa-let-7d-3p**	-1.1482	0.3189	**-1.3241**	**0.0482**	1.1531	0.3044
hsa-let-7d-5p	1.2811	0.2176	1.1769	0.4142	1.0885	0.6695
hsa-let-7e-5p	-1.2326	0.5468	-1.0970	0.7779	-1.1236	0.7229
hsa-let-7f-5p	-1.0123	0.9336	1.1761	0.2737	-1.1905	0.2399
hsa-let-7g-5p	1.0372	0.7420	1.0133	0.9051	1.0236	0.8336
hsa-let-7i-5p	1.0572	0.6233	1.0417	0.7179	1.0149	0.8962
hsa-miR-100-5p	-1.0191	0.9235	-1.3046	0.1828	1.2802	0.2150
hsa-miR-101-3p	-1.0512	0.6910	1.0714	0.5834	-1.1263	0.3468
hsa-miR-103a-3p	1.0865	0.3952	-1.0345	0.7270	1.1240	0.2338
hsa-miR-106a-5p	-1.0588	0.7197	-1.0067	0.9663	-1.0517	0.7514
hsa-miR-106b-5p	1.1647	0.2654	1.0731	0.6035	1.0854	0.5466
hsa-miR-107	1.2169	0.0882	1.1193	0.3202	1.0873	0.4586
hsa-miR-10b-5p	1.1066	0.6047	-1.0840	0.6800	1.1996	0.3548
hsa-miR-122-5p	-1.2356	0.4737	-1.0033	0.9911	-1.2316	0.4806
hsa-miR-125a-5p	-1.0024	0.9919	-1.1136	0.6429	1.1109	0.6269
hsa-miR-125b-5p	1.0534	0.7788	-1.0258	0.8907	1.0805	0.6760
hsa-miR-1260a	1.1600	0.3584	1.1625	0.3389	-1.0021	0.9895
hsa-miR-126-3p	-1.0533	0.6916	1.0028	0.9831	-1.0562	0.6761
hsa-miR-126-5p	-1.0224	0.8769	-1.0818	0.5840	1.0581	0.6939
hsa-miR-128-3p	-1.0807	0.7035	1.1882	0.3999	-1.2841	0.2252
hsa-miR-130a-3p	1.0533	0.7259	-1.0152	0.9187	1.0693	0.6511
**hsa-miR-130b-3p**	1.1923	0.1912	-1.1704	0.2410	**1.3956**	**0.0167**
hsa-miR-132-3p	1.0107	0.9572	1.0919	0.6576	-1.0803	0.6967
hsa-miR-133a-3p	-1.0054	0.9872	-1.4620	0.2615	1.4541	0.2802
hsa-miR-133b	1.0855	0.7571	-1.2405	0.4310	1.3466	0.2793
hsa-miR-136-3p	-1.0755	0.8678	-1.3871	0.4395	1.2897	0.5807
hsa-miR-136-5p	1.0090	0.9848	-1.7809	0.2159	1.7969	0.2211
hsa-miR-139-5p	1.1161	0.7109	1.0317	0.9114	1.0818	0.7797
hsa-miR-140-3p	1.0787	0.5890	1.0371	0.7946	1.0401	0.7788
hsa-miR-140-5p	-1.3120	0.2147	1.1010	0.6567	-1.4445	0.0963
**hsa-miR-141-3p**	**2.2021**	**0.0297**	1.8871	0.0585	1.1669	0.6563
hsa-miR-142-3p	1.0283	0.8386	-1.0117	0.9323	1.0403	0.7729
hsa-miR-142-5p	-1.0288	0.8651	-1.1339	0.4542	1.1022	0.5617
hsa-miR-143-3p	-1.6932	0.1062	-1.2146	0.5433	-1.3941	0.2889
hsa-miR-144-3p	1.1281	0.4163	1.2059	0.2102	-1.0689	0.6518
hsa-miR-144-5p	-1.0864	0.7916	-1.1455	0.6650	1.0544	0.8657
hsa-miR-145-5p	1.1432	0.5586	1.2510	0.3301	-1.0943	0.6932
hsa-miR-146a-5p	-1.2662	0.2661	-1.2057	0.3762	-1.0501	0.8159
hsa-miR-146b-5p	-1.0304	0.9199	1.4749	0.2272	-1.5197	0.1830
hsa-miR-148a-3p	-1.1975	0.4461	-1.1172	0.6385	-1.0719	0.7620
hsa-miR-148b-3p	-1.0374	0.7245	-1.0773	0.4758	1.0385	0.7167
hsa-miR-150-5p	1.0558	0.7063	-1.0768	0.6079	1.1369	0.3758

hsa-miR-151a-3p	-1.3826	0.1610	-1.2583	0.3164	-1.0988	0.6791
hsa-miR-151a-5p	1.0034	0.9800	1.0155	0.9107	-1.0121	0.9306
hsa-miR-152-3p	-1.3902	0.0998	-1.2320	0.2909	-1.1284	0.5384
hsa-miR-154-5p	-1.5618	0.2451	-1.8954	0.0999	1.2136	0.6284
**hsa-miR-15a-5p**	**1.3380**	**0.0257**	1.2109	0.1335	1.1050	0.4277
hsa-miR-15b-3p	-1.0435	0.8213	1.1590	0.4234	-1.2094	0.3169
hsa-miR-15b-5p	-1.0125	0.9416	1.0415	0.8103	-1.0545	0.7542
hsa-miR-16-2-3p	1.1075	0.5347	1.3374	0.0837	-1.2076	0.2551
hsa-miR-16-5p	1.0632	0.7163	-1.0655	0.7068	1.1328	0.4610
hsa-miR-17-5p	-1.0343	0.9066	-1.1152	0.6966	1.0783	0.7929
hsa-miR-181a-5p	1.0119	0.9530	-1.1579	0.4533	1.1716	0.4306
hsa-miR-185-5p	-1.0150	0.9141	1.1011	0.4868	-1.1176	0.4227
hsa-miR-186-5p	2.1966	0.0570	1.1500	0.7338	1.9101	0.1135
hsa-miR-18a-5p	-1.3951	0.2844	-1.2186	0.5226	-1.1449	0.6611
hsa-miR-18b-5p	-1.1187	0.6090	1.2518	0.3088	-1.4004	0.1309
hsa-miR-191-5p	1.2425	0.0658	1.0946	0.4206	1.1352	0.2737
hsa-miR-192-5p	-1.2042	0.4113	-1.1117	0.6383	-1.0832	0.7227
hsa-miR-194-5p	1.0540	0.7751	-1.2473	0.2355	1.3147	0.1443
hsa-miR-195-5p	1.0647	0.8683	1.4548	0.3102	-1.3664	0.4125
hsa-miR-197-3p	-1.2128	0.4009	-1.5315	0.0627	1.2627	0.3112
hsa-miR-199a-3p	-1.1962	0.4120	-1.2186	0.3659	1.0187	0.9319
hsa-miR-199a-5p	-1.1090	0.7817	1.1137	0.7790	-1.2351	0.5829
hsa-miR-19a-3p	-1.0618	0.6599	1.0796	0.5746	-1.1463	0.3196
hsa-miR-19b-3p	-1.1166	0.4827	1.0017	0.9911	-1.1185	0.4759
hsa-miR-205-5p	1.2026	0.6137	1.1591	0.6859	1.0375	0.9174
hsa-miR-20a-5p	1.0294	0.8405	-1.0179	0.9019	1.0478	0.7456
hsa-miR-210-3p	-1.1524	0.4854	1.1235	0.5663	-1.2946	0.2081
hsa-miR-2110	-1.1708	0.6456	1.0847	0.8070	-1.2699	0.4746
hsa-miR-215-5p	-1.2474	0.4525	-1.1019	0.7406	-1.1321	0.6724
hsa-miR-21-5p	1.0175	0.8547	1.0154	0.8714	1.0020	0.9830
hsa-miR-221-3p	-1.1247	0.4778	-1.1840	0.3096	1.0527	0.7553
hsa-miR-222-3p	1.0411	0.6730	-1.0671	0.4971	1.1109	0.2742
hsa-miR-223-3p	-1.2428	0.2548	-1.2995	0.1718	1.0456	0.8132
hsa-miR-22-3p	1.0319	0.7949	1.0977	0.4420	-1.0638	0.6091
hsa-miR-22-5p	1.1488	0.4831	1.2187	0.3194	-1.0608	0.7645
hsa-miR-23a-3p	-1.1289	0.3751	-1.1705	0.2517	1.0368	0.7903
hsa-miR-23b-3p	-1.0995	0.5057	-1.1236	0.4146	1.0219	0.8789
hsa-miR-24-3p	-1.3203	0.0735	-1.2808	0.1090	-1.0309	0.8406
hsa-miR-25-3p	1.0667	0.6517	1.0422	0.7725	1.0235	0.8707
hsa-miR-26a-5p	1.1012	0.6059	1.1478	0.4616	-1.0423	0.8241
**hsa-miR-26b-5p**	**1.6245**	**0.0094**	1.0833	0.6511	**1.4996**	**0.0276**
hsa-miR-27a-3p	-1.1738	0.4534	-1.1518	0.5082	-1.0192	0.9289
hsa-miR-27b-3p	-1.1181	0.5953	-1.0005	0.9980	-1.1175	0.5970
hsa-miR-28-3p	1.0345	0.9070	1.3420	0.3275	-1.2972	0.3591
hsa-miR-29a-3p	-1.1971	0.1837	1.0609	0.6580	-1.2700	0.0805
hsa-miR-29b-3p	1.0367	0.8108	1.0879	0.5765	-1.0494	0.7488
hsa-miR-29c-3p	1.0924	0.5043	1.1178	0.4011	-1.0232	0.8617
hsa-miR-301a-3p	1.3881	0.4695	1.0932	0.8358	1.2698	0.5647
hsa-miR-30a-5p	-1.4192	0.2197	-1.3318	0.3132	-1.0656	0.8216

hsa-miR-30b-5p	-1.0957	0.5525	1.0406	0.7955	-1.1401	0.3953
hsa-miR-30c-5p	-1.1927	0.2556	-1.1283	0.4334	-1.0570	0.7178
**hsa-miR-30d-5p**	-1.1674	0.1514	**-1.2399**	**0.0496**	1.0621	0.5712
hsa-miR-30e-3p	1.8872	0.2514	1.2211	0.7049	1.5455	0.4406
hsa-miR-30e-5p	-1.0058	0.9598	1.0379	0.7462	-1.0439	0.7085
hsa-miR-320a	1.1865	0.1176	-1.0042	0.9689	1.1915	0.1093
hsa-miR-320b	1.2052	0.1602	1.1081	0.4347	1.0876	0.5221
hsa-miR-320c	1.3638	0.1341	1.3826	0.1184	-1.0138	0.9463
hsa-miR-324-3p	1.2836	0.1827	1.3872	0.0838	-1.0807	0.6748
**hsa-miR-324-5p**	**-2.1265**	**0.0021**	**-1.6679**	**0.0216**	-1.2750	0.2856
hsa-miR-32-5p	1.1478	0.2491	1.1602	0.2150	-1.0108	0.9279
hsa-miR-326	-1.3251	0.3268	-1.1563	0.6106	-1.1459	0.6125
**hsa-miR-328-3p**	**-1.8574**	**0.0199**	-1.5722	0.0826	-1.1814	0.5135
hsa-miR-331-3p	-1.0257	0.8553	1.0665	0.6438	-1.0939	0.5200
**hsa-miR-335-5p**	**-2.3264**	**0.0053**	-1.6267	0.0939	-1.4301	0.2132
hsa-miR-338-3p	-1.2225	0.4547	-1.4750	0.1534	1.2066	0.4954
**hsa-miR-339-3p**	1.7232	0.0965	-1.1122	0.7256	**1.9165**	**0.0390**
hsa-miR-339-5p	-1.1867	0.6243	-1.0029	0.9934	-1.1833	0.6388
hsa-miR-33a-5p	-1.4070	0.3587	-1.4556	0.3136	1.0345	0.9268
hsa-miR-342-3p	1.0312	0.8499	-1.0530	0.7505	1.0859	0.6125
hsa-miR-34a-5p	1.3709	0.2557	-1.0299	0.9166	1.4119	0.2149
hsa-miR-361-5p	-1.4944	0.0786	-1.3283	0.2081	-1.1250	0.5974
hsa-miR-362-3p	-1.2372	0.1440	-1.0637	0.6666	-1.1631	0.2955
hsa-miR-363-3p	-1.0262	0.8771	-1.0506	0.7681	1.0237	0.8884
**hsa-miR-365a-3p**	-1.4961	0.1704	**-2.2932**	**0.0058**	1.5328	0.1469
hsa-miR-374a-5p	-1.0148	0.9612	-1.1324	0.6892	1.1159	0.7243
**hsa-miR-374b-5p**	-1.0743	0.7562	**-1.8096**	**0.0181**	**1.6844**	**0.0399**
hsa-miR-375	-1.2800	0.5808	-1.1204	0.7935	-1.1425	0.7653
hsa-miR-376a-3p	-1.4297	0.2995	-1.5587	0.1882	1.0902	0.8003
**hsa-miR-376c-3p**	**-2.1877**	**0.0439**	**-2.1192**	**0.0468**	-1.0323	0.9324
**hsa-miR-378a-3p**	**-1.4790**	**0.0266**	-1.1593	0.3856	-1.2757	0.1573
hsa-miR-382-5p	-1.3112	0.3954	-1.2607	0.4666	-1.0401	0.9063
hsa-miR-421	-1.5363	0.2323	1.0512	0.8802	-1.6150	0.1839
hsa-miR-423-3p	-1.0548	0.7989	1.0413	0.8468	-1.0984	0.6546
hsa-miR-423-5p	1.2986	0.0796	1.2290	0.1625	1.0566	0.7050
**hsa-miR-424-5p**	-1.0526	0.8482	1.6552	0.0670	**-1.7422**	**0.0448**
hsa-miR-425-3p	-1.1487	0.4331	-1.1391	0.4469	-1.0084	0.9629
hsa-miR-425-5p	1.0621	0.6548	-1.0276	0.8393	1.0914	0.5168
hsa-miR-451a	1.2293	0.1450	1.0936	0.5219	1.1241	0.4032
hsa-miR-454-3p	1.4116	0.3801	1.1829	0.6593	1.1934	0.6426
hsa-miR-483-5p	-1.4457	0.4409	-1.0599	0.9027	-1.3640	0.5372
hsa-miR-484	1.0033	0.9789	-1.1200	0.3719	1.1237	0.3582
**hsa-miR-486-3p**	1.7020	0.0529	**2.1593**	**0.0098**	-1.2687	0.3727
hsa-miR-486-5p	1.2754	0.1163	1.2278	0.1826	1.0388	0.7966
hsa-miR-497-5p	1.3049	0.3117	1.0041	0.9876	1.2996	0.3191
**hsa-miR-501-3p**	-1.3106	0.4055	**-2.3638**	**0.0171**	1.8037	0.0933
hsa-miR-502-3p	1.0430	0.8249	1.1918	0.3597	-1.1427	0.4851
hsa-miR-505-3p	1.3100	0.3069	1.0342	0.8915	1.2666	0.3701
hsa-miR-532-3p	-1.1444	0.5387	-1.5293	0.0593	1.3363	0.1912

hsa-miR-532-5p	-1.0018	0.9932	-1.0172	0.9373	1.0153	0.9440
hsa-miR-574-3p	-1.2094	0.5026	1.0347	0.9040	-1.2514	0.4028
hsa-miR-584-5p	-1.3340	0.3144	-1.0921	0.7568	-1.2216	0.4829
hsa-miR-590-5p	1.1412	0.3727	1.2116	0.1984	-1.0617	0.6846
hsa-miR-629-5p	1.0296	0.9119	1.0109	0.9673	1.0185	0.9445
hsa-miR-652-3p	-1.0059	0.9663	-1.0902	0.5386	1.0838	0.5667
hsa-miR-660-5p	1.1772	0.2590	-1.0260	0.8576	1.2078	0.1930
hsa-miR-7-5p	-1.0020	0.9950	1.0406	0.8967	-1.0427	0.8984
hsa-miR-766-3p	1.0088	0.9748	-1.4056	0.2148	1.4179	0.2035
hsa-miR-874-3p	1.1513	0.5149	1.1880	0.4141	-1.0319	0.8840
hsa-miR-877-5p	1.1520	0.6451	1.0035	0.9904	1.1480	0.6532
hsa-miR-885-5p	-1.0479	0.9108	-1.1310	0.7686	1.0792	0.8553
hsa-miR-92a-3p	1.0901	0.3939	-1.0237	0.8158	1.1160	0.2801
hsa-miR-93-5p	1.0019	0.9900	-1.0294	0.8468	1.0314	0.8370
hsa-miR-99a-5p	-1.0703	0.7266	-1.2451	0.2637	1.1633	0.4380
hsa-miR-99b-5p	1.3726	0.4537	1.2247	0.6213	1.1208	0.7865

FC = fold change. NG = normoglycemia; GI = glucose intolerant; DM = newly diagnosed diabetes. Parameters with significant differences are indicated in bold.

Suppl. 5. Immunoblotting images for anti-CD9, anti-CD81, and anti-HSP70 of four random plasma-derived extracellular vesicle samples.

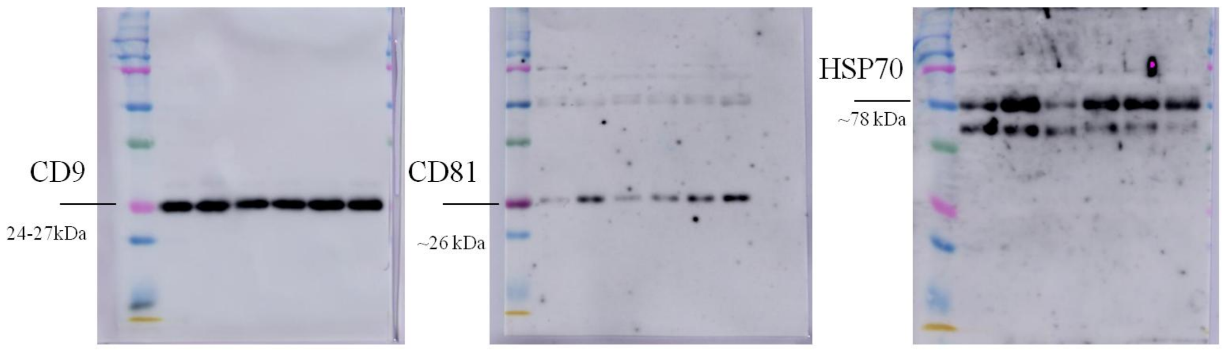

Supplement: Supplementary file 1 — AppendixS1 [file PHY2-9-e14731-s001.pdf]
